# Alcohol Consumption, Progression of Disease and Other Comorbidities, and Responses to Antiretroviral Medication in People Living with HIV

**DOI:** 10.1155/2012/751827

**Published:** 2012-03-11

**Authors:** Manuela G. Neuman, Michelle Schneider, Radu M. Nanau, Charles Parry

**Affiliations:** ^1^Departments of Pharmacology & Toxicology and Global Health, University of Toronto, Toronto, ON, Canada M5S 1A1; ^2^In Vitro Drug Safety and Biotechnology, MaRS Discovery District, 101 College Street, Suite 300, Lab 351, Toronto, ON, Canada M5G 1L7; ^3^Institute of Drug Research, Canada; ^4^Alcohol and Drug Abuse Research Unit, South African Medical Research Council, Cape Town-Tygerberg 7505, South Africa; ^5^Department of Psychiatry, Stellenbosch University, Cape Town-Tygerberg 7505, South Africa

## Abstract

The present paper describes the possible connection between alcohol consumption and adherence to medicine used to treat human deficiency viral (HIV) infection. Highly active antiretroviral therapy (HAART) has a positive influence on longevity in patients with HIV, substantially reducing morbidity and mortality, including resource-poor settings such as South Africa. However, in a systematic comparison of HAART outcomes between low-income and high-income countries in the treatment of HIV-patients, mortality was higher in resource-poor settings. Specifically, in South Africa, patients often suffer from concomitant tuberculosis and other infections that may contribute to these results. Alcohol influences the use of medicine for opportunistic infections (e.g., pneumonia, tuberculosis), or coinfections HIV-hepatitis viruses-B (HBV) and C (HCV), cytomegalovirus, or herpes simplex virus. Furthermore, alcohol use may negatively impact on medication adherence contributing to HIV progression. The materials used provide a data-supported approach. They are based on analysis of published (2006–2011) world literature and the experience of the authors in the specified topic. Intended for use by health care professionals, these recommendations suggest approaches to the therapeutic and preventive aspects of care. Our intention was to fully characterize the quality of evidence supporting recommendations, which are reflecting benefit versus risk, and assessing strength or certainty.

## 1. Introduction

Failure to recognize alcohol behaviour remains a significant problem that impairs efforts directed towards the prevention and management of patients with alcoholic liver damage. Although there are limitations in the available data, the World Health Organization's Global Alcohol database, which has been in existence since 1996, has been used to estimate worldwide patterns of alcohol consumption, and it allows comparisons of alcohol-related morbidity and mortality. The burden of alcohol-related disease is highest in the developing world, including South Africa. Pithey and Parry [1] describe the association between alcohol use and human immunodeficiency virus (HIV) infection in a systematic review of sub-Saharan African studies. The authors present studies that have quantified the association between alcohol consumption and HIV infection in this region. They analyzed work performed between 2000 and 2008 that reported relative measures of the association between alcohol use and HIV prevalence and/or seroconversion rates. However, the authors sustain that in order to confirm causality, the use of clearly defined standardised measures of alcohol use is needed [1]. Patterns of alcohol consumption are expressed and regulated differently in diverse geographical regions. There are contradictory drinking guidelines defining low-risk and high-risk drinking in different countries. In the United States of America, the National Institute of Alcohol and Alcohol Abuse (NIAAA) and the United States Department of Agriculture define low risk drinking as ≤14 drinks/week and ≤4 drinks on any day for men. For women, the definition of low risk drinking is ≤7 drinks/week or ≤3 drinks on any day (http://www.rethinkingdrinking.niaaa.nih.gov/; http://www.cnpp.usda.gov/dgas2010-dgacreport.htm). Proposed guidelines specific for each nation make it difficult to conduct an international generalization of “moderate, low-risk drinking” versus “high-risk drinking.”

## 2. Material and Methods

We performed a systematic review of published PubMed literature, searching for articles that contained information about “alcohol”, “HIV” and “antiretroviral therapy” published between January 2006 and June 2011. We did not limit our search to literature published in English. We found over 365 results using the key words “alcohol,” “adherence,” “ART” and “HIV,” from which we selected 230 articles that we analyzed.

From these initial results, we selected 25 articles to be included in the “disease progression” sections and 38 articles to be included in the “adherence” section. Particular attention was placed on those papers that provided an indication of the type and the amount of alcohol consumed. In order to obtain more focused results so that we could, where necessary, refer to South Africa, we also included the words “South Africa” in the search. However, we did not have “South Africa” as an exclusion criterion. Main reasons for excluding articles include poor characterization of alcohol consumption patterns, incomplete or poor characterization of adherence to medication or/and disease progression, *in vitro* or* in vivo* animal studies, and studies where the focus was on comorbid diseases and addictions, as well as treatments for these conditions, whose effects could have undermined that of alcohol (e.g., environmental habits (drugs of abuse, smoking), viral infections (cytomegalovirus, herpes simplex virus, hepatitis C, hepatitis B), malaria, tuberculosis).

Although not specific for the main topics discussed, some relevant papers published prior to 2006 contained important information that was used to reinforce our arguments and were therefore discussed as well.

**Figure 1:**
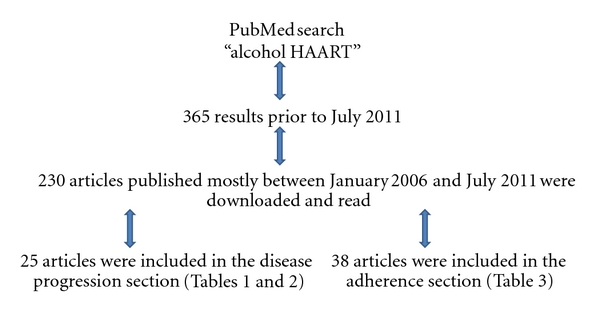
Methods used for literature search.

Figure 1 illustrates the methods used for the literature search and the number of articles chosen for different subjects. Data accessible in this paper are descriptive in nature. All prevalence estimates of alcohol use are the data presented by their respective authors.

## 3. Results and Discussion

### 3.1. HIV and Alcohol Misuse

Rehm and Parry [2] described the link between alcohol consumption and infectious diseases in South Africa. Alcohol abuse is often associated with numerous facets of HIV disease progression, ranging from hepatotoxicity to immune system impairment.  Table 1 presents the role played by alcohol on the progression of HIV-associated disease symptoms.

Neuman et al. [3], Núñez [4], and Barve et al. [5] extensively reviewed hepatotoxicity associated with alcohol use and highly active antiretroviral therapy (HAART) administration. The development of lung infection was reviewed by Rehm et al. [6] and Quintero and Guidot [7], while the progress of cardiovascular diseases was reviewed by Freiberg and Kraemer [8]. Rosenbloom et al. [9] reviewed detrimental effects on the structure, chemistry, and function of the central nervous system.

Neuman et al. [3] discuss the interactions between therapeutic drugs used to minimize and control drug and alcohol dependence. Furthermore, drug-drug interactions occur between HAART and alcohol, different HAART components and methadone, or each one of the therapies with the other, thus contributing to a higher toxicity level. With the evolution of effective antiretroviral therapy (ART), survival of persons living with HIV and acquired immunodeficiency syndrome (AIDS) has increased dramatically, leading to more interactions with other liver related comorbidities such as alcohol and viral hepatitis and the drugs used to treat these diseases.

The following section will review several studies that analyzed the relationship between alcohol misuse and HIV disease progression. Two important laboratory determinants of the rate of disease progression are the CD4^+^ cell counts and the plasma viral load.

#### 3.1.1. Role of Hepatitis Viruses on HIV

Hazardous drinking is often associated with liver disease [10, 11], particularly among hepatitis C virus (HCV) monoinfected patients and HIV/HCV coinfected patients [12]. Several interesting trends were observed in the MORTAVIC study, a multicentre prospective cross-sectional survey of French hospital departments of internal medicine and infectious diseases participating in the treatment of HIV-infected individuals [13, 14]. From 215 deaths that occurred in 2003 among 20940 HIV positive, 27 (12.6%) can be attributed to end-stage liver disease. Of these, HCV coinfection was present in 25 (92.6%) patients, alcohol consumption of any kind in 25 (92.6%) patients, moderate alcohol consumption (30–60 g/day) in 12 (44.4%) patients, and heavy alcohol consumption (>60 g/day) in 7 (26.0%) patients [13]. Over the previous decade, the proportion of patients dying from AIDS decreased and the number of patients dying from end-stage liver disease remained relatively constant. In recent times, the proportion of patients dying from end-stage liver disease is significantly higher (21 out of 1426 deaths (1.5%) in 1995 versus 27 out of 215 (12.6%) deaths in 2003, *P* < 0.01). Among patients dying from end-stage liver disease, the proportion of patients with HCV coinfection alone and the proportion of excessive alcohol consumption were significantly higher in 2003 compared to 1995 [13].

From 287 deaths that occurred in 2005 among 24000 HIV positive patients followed at multiple centers in France, 48 (16.7%) can be attributed to end-stage liver disease [14]. Of these, hepatitis virus coinfection was present in 45 (93.8%) patients, with 38 (79.2%) patients suffering from HCV coinfection. Excessive alcohol consumption (>30 g/day) was reported by 23 (47.9%) patients in this subsequent study [14]. Alcohol consumption was related to death in 4 HCV/HIV coinfected patients (10.5%), while HCV coinfection led to an additional 8 deaths (21.0%) in HIV positive patients who abused alcohol, as assessed by the patients' physicians. An additional case of lethal cirrhosis was identified independent of alcohol consumption or viral hepatitis coinfection [14]. Overall, 36 (75.0%) patients died from cirrhosis, 7 (14.6%) patients died from HCV coinfection and 5 (10.4%) patients died from hepatitis B virus (HBV) coinfection. Hepatitis virus coinfection (*P* < 0.001) and consuming alcohol in excess of 30 g/day (*P* = 0.005) were significantly associated with death due to end-stage liver disease [14].

#### 3.1.2. Role of Cirrhosis in Disease Progression

While both HIV positivity and excessive drinking were independently associated with cirrhosis, the proportion of patients with cirrhosis was higher in HIV positive individuals (18/60, 30.0%), compared to HIV-negative individuals (23/150, 15.3%) (*P* < 0.0001) in another French study [15]. There were no differences in the incidence of cirrhosis between HIV positive excessive drinkers and HIV-negative excessive drinkers. This should be interpreted with care, as the low number of patients included in this study, particularly HIV positive patients, could prevent the identification of an interaction between HIV positivity and excessive drinking with respect to the development of cirrhosis [15].

Among 181 cases of liver cirrhosis in a large sample of 2168 HIV positive patients, 149 (82.3%) were caused by HCV, 3 (1.6%) were caused by HBV, 5 (2.8%) were caused by dual HBV/HCV coinfection, and 12 (6.6%) were caused by triple HBV/HCV/hepatitis D virus coinfection [16]. Alcohol consumption, significantly associated with a diagnosis of cirrhosis, was found to be more frequent among patients with chronic viral hepatitis compared to patients without these coinfections (*P* < 0.001). Interestingly, alcohol was not found to be the only cause of cirrhosis in any one patient [16]. Aside from high alcohol consumption, coinfection with HCV and/or HBV are risk factors for developing liver toxicity (OR 10.36, 95% CI 1.38–77.56, *P* = 0.03) [17].

#### 3.1.3. Alcohol-Induced Inflammation Leads to Progression of HIV and Other Comorbid Reactions

Many processes related to the consumption or breakdown of alcohol that contribute to alcohol-induced liver disease are mediated by small proteins known as cytokines, which are produced and secreted by liver cells and many other cells throughout the body [18]. Through a variety of actions, cytokines regulate certain biochemical processes in the cells that produce them, as well as in neighbouring cells. For example, in the case of HIV infection, they attract white blood cells to the tissue, triggering an inflammatory response. In the liver, persistent cytokine secretion resulting in chronic inflammation leads to conditions such as hepatitis, fibrosis and cirrhosis. Cytokines also regulate a process known as programmed cell death, or apoptosis, which is in part responsible for alcohol-induced loss of liver tissue [19].

Dyslipidemia, consisting of hypertriglyceridemia, low high-density lipoprotein (HDL) cholesterol, and elevated low-density lipoprotein (LDL) cholesterol, is being observed with increasing frequency among persons living with HIV. Hazardous alcohol consumption, particularly among Hispanic individuals and in individuals consuming the highest amounts of alcohol, worsens dyslipidemia [20].

Alcohol use and not being on HAART (*P* < 0.001) are independent predictors of pneumonia in HIV positive smokers [21]. The incidence of pneumonia was significantly lower in the HAART era compared to the pre-HAART era (*P* < 0.01), although alcohol abuse remains an independent risk factor for developing bacteremic pneumococcal disease [22].

Cerebrovascular ischemia was associated with a history of high alcohol intake, and fewer months on HAART (OR 0.97, 95% CI 0.96–0.99; *P* < 0.001). This suggests that long-term HAART has a protective effect against cerebrovascular ischemia, yet this effect is countered by a history of alcohol abuse [23].

A history of alcohol abuse or dependence was not associated with neuropathic pain caused by HIV-associated sensory neuropathy [24].

Significant differences were found between HIV positive heavy drinkers and HIV-negative light drinkers with respect to motor and visuomotor speed, pointing to a synergistic interaction between alcohol abuse and HIV infection [25].

Impaired upper limb function was observed between clinical groups (HIV positive patients, patients with alcoholism, and patients with HIV infection and alcoholism comorbidity) and controls in terms of upper motor composite score (*P* = 0.008 for HIV group, *P* = 0.031 for alcoholism groups and *P* = 0.003 for HIV and alcoholism comorbidity group) and slower fine finger movement (*P* = 0.004 for HIV group, *P* = 0.033 for alcoholism groups and *P* = 0.0003 for HIV and alcoholism comorbidity group) [26]. Although not significant, HIV and alcoholism comorbidity impair upper motor limb function to a greater degree that HIV alone or alcoholism alone. There were significant differences between groups with respect to closed eye composite scores (stand heel-to-toe, walk heel-to-toe, and stand on one leg with eyes closed tasks) (*P* = 0.013) [26]. These differences could not be explained by the presence of peripheral neuropathy, HAART, or AIDS-defining events [26].

Immediate episodic memory was found to be impaired in HIV positive patients suffering from alcoholism, compared to either HIV positive patients without a drinking problem, HIV-negative patients suffering from alcoholism or normal controls [27]. Interestingly, these results could not be explained by the amount of alcohol consumed over a lifetime, CD4^+^ cell counts, AIDS diagnosis, or HAART medication. HIV infection or alcoholism alone did not affect immediate episodic memory. Also, working memory and the ability to retain information over time were not impaired by HIV infection or alcoholism [27].

Alcohol abuse was not associated with a longer time before clinical stability was achieved among patients who developed bacterial community-acquired pneumonia [28].

#### 3.1.4. Nonalcoholic Steatohepatitis and HIV Disease Progression

While alcohol abuse is generally associated with HIV disease progression, several studies did not find such an association. For example, Crum-Cianflone et al. [29] found that the most common diagnosis among HIV positive patients with liver test abnormalities was that of non-alcoholic fatty liver disease. The amount of alcohol consumed per week and alcohol abuse were not predictors of liver test abnormalities. The low number of patients suffering from viral hepatitis coinfection was not high enough to uncover any effect of these comorbidities. While ART use overall did not predict liver test abnormalities, the use of protease inhibitors did (*P* = 0.04) [29]. A separate study found that alcohol consumption was not associated with HCV-related serious adverse reactions in a cohort of 1175 HIV-infected patients (1048 (89.2%) were HCV coinfected) [30].

#### 3.1.5. Injecting Drug Users and HIV

Compared to healthy noninjecting drug users (IDU), HIV patients who were not alcohol abusers (control population), HIV IDU only, HIV alcohol abusers, and IDU alone were each significantly associated with a lower level of CD4^+^ lymphocyte recovery (*P* < 0.04) [31]. However, no such association was found with respect to alcohol abuse alone. Compared to patients who did not abuse either alcohol or injectable drugs, no significant differences in terms of virological response (i.e., undetectable viral load) were found for either of the three study groups [31]. Unfortunately, the effects of alcohol consumption on HAART adherence are not analyzed in this study [31].

### 3.2. The Role of Alcohol Consumption on the Immune System and the HIV Viral Load

Table 2 presents some recent data on the role played by alcohol on CD4^+^ cell counts and the plasma viral load.

Alcohol abuse after contacting HIV seems to accelerate disease progression through a direct effect on CD4^+^ cells. Of note is the detrimental role played by alcohol consumption on CD4^+^ cell counts, particularly among individuals not on ART [32–36]. Heavy alcohol consumption is associated with a four times lower chance of achieving undetectable viral load and a two times higher chance of having low CD4^+^ cell counts, compared to moderate alcohol consumption or abstinence [33]. Alcohol is an immunosuppressant acting directly through T-cell apoptosis, mitochondrial damage, and inhibition of T-cell responses, natural killer cell activity and macrophage phagocytic activity. Alcohol consumption may increase susceptibility to opportunistic infections and accelerate disease progression among HIV positive individuals. Additionally, alcohol leads to impaired viral load response and reduced CD4^+^ cell reconstitution [35]. Frequent alcohol use is significantly associated with low CD4^+^ cell counts and higher viral loads over time [35].

In patients not on ART, heavy alcohol consumption was associated with lower CD4^+^ cell counts compared to patients with a history of abstinence. At-risk drinkers (4 drinks/week for women and 5 drinks/week for men) were less likely to have a current HAART prescription (*P* < 0.05) and were less likely to have suppressed viremia if they had a current HAART prescription (*P* < 0.05), compared to nondrinkers [36]. Consuming more than 5 drinks/week is a predictor for not being on HAART and for having an unsuppressed viral load [36]. Moreover, the risk of opportunistic infections increases as CD4^+^ cell counts decline.

The type of alcohol being consumed is important with regards to outcome in HIV positive patients currently taking HAART [37]. In subjects consuming only beer or wine, increases in thymus size and in CD4^+^ cell counts were observed following HAART initiation. In contrast, consumption of only liquor was associated with decreases in both thymus size and in CD4^+^ cell counts, particularly evident in women. Míguez-Burbano et al. [37] conclude that liquor consumption is associated with thymus deterioration and poor virologic and immunologic control in HIV positive patients taking ART.

Moderate alcohol use (<1 drink per day for the past 6 months) did not significantly increase the rate in CD4^+^ cell count decline to ≤200 cells/*μ*L, compared to abstainers. Frequent alcohol use (>2 drinks/day) resulted in a risk of CD4^+^ cell counts decline that was almost three times higher than that for moderate alcohol use. CD4^+^ cell counts decline was faster in frequent alcohol users who were not on ART than in those who were on ART [34, 35]. CD4^+^ cell counts decline was faster in frequent alcohol users who combined alcohol with crack cocaine. Viral load was found to be 0.259 log10 units higher in frequent alcohol users than in moderate alcohol users and abstainers. This relationship was found to be significant in patients who were receiving ART. Alcohol use had no impact on HIV viral loads in patients not receiving ART. One possible explanation for these findings in that heavy alcohol use is deleterious in patients on ART because it might decrease patient adherence to ART, rather than alcohol having a direct effect on viral load [34, 35]. A drop in CD4^+^ cell counts is mediated by the direct toxic effect of alcohol on these lymphocytes, which appears to be independent of the viral load. At the same time, Samet et al. [34] argue that the beneficial effect of ART on CD4^+^ cell counts may account for a lower toxicity seen when alcohol is abused in the presence of ART.

Consuming ≥8 alcoholic drinks/week was related to a higher risk of death [38]. In fact, Braithwaite et al. [39] found that alcohol consumption of any kind decreases survival in HIV positive patients.

### 3.3. The Role of Alcohol Consumption on Medication Adherence

Drug and/or alcohol abuse and suboptimal ART adherence are predictors of virological failure [40].  Table 3 presents studies in which alcohol consumption modulates medication adherence.

Following the introduction of HAART in 1996, individuals living with HIV taking this form of medication have benefited from improvements in immunological and virological parameters, as well as an improved quality of live and longevity [41]. However, adherence to HAART in excess of 95% is often regarded as optimal in order to benefit from this treatment [41].

#### 3.3.1. Alcohol Consumption and Nonadherence to ART

Numerous studies from around the world document the detrimental effect of alcohol on HAART adherence, from the United States [36, 42–55], to Europe [56–61], Australia [62], Africa [63–69], South America [70, 71], and Asia [72, 73]. Alcohol consumption is associated with the first nonstructured treatment interruption, early (within the first year) versus late treatment interruption, and interruption of longer duration (≥6 months) [53, 66].

In addition to nonadherence, at-risk drinkers were less likely to have a current HAART prescription. As a result, at-risk drinking was a predictor of not being on HAART [36, 51]. In fact, all levels of drinking were associated with higher odds of not using HAART compared to alcohol abstinence [54], such that dose-dependent worsening of adherence was found with increasing alcohol consumption [49, 54, 60]. The highest degree of nonadherence was found in cases where alcohol use was classified as problem drinking (defined as meeting NIAAA criteria for at-risk drinking or diagnostic criteria for an alcohol use disorder) (OR 0.474, 95% CI 0.408–0.550), while it was lower in studies examining any or global alcohol use (OR 0.604, 95% CI 0.531–0.687) [41]. In the combined analysis of 40 studies reviewed in a meta-analysis, alcohol drinkers were approximately 50–60% as likely to be classified as adherent (OR 0.548, 95% CI 0.490–0.612) compared with abstainers (or those who drank relatively less) [41].

Concurrent crack cocaine use is associated with even lower adherence (OR 3.61, 95% CI 1.56–8.35, *P* < 0.01) [51], as is a lifetime history of being an IDU (OR 2.17, 95% CI 1.16–4.05, *P* = 0.015) [70].

Nonadherence is often associated with unsuppressed viremia. For example, Shacham et al. [36] found that consuming more than 5 drinks/week is a predictor for having an unsuppressed viral load.

The reasons behind the association between alcohol consumption and nonadherence are varied. For example, due to the belief that alcohol should not be mixed with their medication, people living with HIV/AIDS may interrupt their medication when they are drinking [52] or delay HIV treatment while trying to cope with alcohol dependence [64]. Forgetfulness does seem to play an important role, as substance use by the caregiver was associated with higher odds of ART nonadherence among children in their care [42, 65]. Alcohol also appears to affect adherence to ART through conscious decisions to skip medication while drinking and not through drunken forgetfulness [45]. Based on their research, Sankar et al. [45] found that light drinkers are the most likely subgroup to miss medication.

#### 3.3.2. Other Factors Linked to Nonadherence

Drinking patterns were found to differ across gender and ethnic groups. For example, hazardous drinking was more predominant among African-American (*P* < 0.01) and mixed race (*P* < 0.04) patients, compared to white patients, and African American patients were less likely to report 100% adherence (OR 0.35, 95% CI 0.17–0.71, *P* < 0.01) [55]. Afrodescent was marginally associated with poor adherence in a Brazilian study as well (OR 1.55, 95% CI 0.97–2.47, *P* = 0.068) [70]. The detrimental effects of alcohol on medication adherence seem to affect women to a greater degree than men [44, 50].

Several other factors are also related to nonadherence. Each additional year of life was associated with further decrease in adherence (OR 0.96, 95% CI 0.92–1.00, *P* < 0.04), while a higher level of medication-specific social support (e.g., companionship or assistance) diminished the negative effects of alcohol consumption on ART adherence (OR 1.06, 95% CI 1.01–1.12, *P* = 0.01) [55].

Significance of alcohol consumption diminishes once stress is factored in, suggesting that life stress may be one of the main causes for alcohol and drug consumption in HIV positive individuals, and alcohol consumption may in turn lead to nonadherence [47].

An interesting observation reported in a South African study is that many participants refused to disclose their HIV status to their family out of fear that their family would consume alcohol as result of such news, highlighting the wide-spread alcohol consumption in some communities [63].

Based on findings from these studies, it is recommended that HIV treatment programs address at-risk drinking as well [51]. The clinical evaluation of a person living with HIV should also determine the prevalence of alcohol use and/or the presence of alcohol use disorders. Moreover, an assessment of concomitant drug and alcohol use, as well as comorbidities, is needed in both men and women.

There have also been reports of no association between alcohol consumption and delayed HAART initiation [74], HAART administration [29], and nonadherence [75], and even lower alcohol consumption among individuals receiving HAART [76] and earlier presentation for HAART initiation among patients consuming alcohol [77]. However, while important, such reports are relatively rare, and there is an overall considerable and consistent association between alcohol consumption and medication nonadherence [41].

#### 3.3.3. ART Adherence and the Development of Class-Specific Medication Resistance

The association between ART adherence and the development of class-specific ART resistance represents a clinical problem. During multidrug therapy, differential drug exposure increases the likelihood of developing resistance. In addition, ART with higher potency and higher genetic barriers to resistance decrease the incidence of resistance for companion ART at all adherence levels. Drug resistance mutations proliferate under conditions of nonsuppressive ART, which is usually the result of inadequate drug exposure [78]. As poor adherence is the major determinant of inadequate drug exposure, ART adherence is critically linked to the development of medication resistance. In low-income countries such as South Africa where alcohol consumption is very high, nonadherence is more often producing resistance mutations, therefore leading to inadequate suppression of the HIV virus. The present review concords with the systematic review of Shuper et al. [79], bringing evidence that alcohol affects the immune system, consequently contributing to a deteriorating course of HIV disease. In addition, alcohol misuse impacts on medication adherence.

## 4. Conclusions

The primary goal of ART is to increase disease-free survival through suppression of viral replication and improvement in immunological function. The optimal time to initiate treatment is influenced by these known benefits and the risk of drug toxicity, potential emergence of viral drug resistance, and the need for lifetime therapy. The complexities of adherence-resistance relationships are related to characteristics of the virus, the medication, misuse of alcohol, and their interactions. Nevertheless, the effectiveness of ART can be limited by lack of access to therapy. Additionally, a set of acquired behaviour, such as alcohol misuse and poor adherence and/or intolerance, can lead to ART resistance. Therefore, especially in low-income populations, the education of individuals who live with HIV and alcohol abuse is relevant.

Knowledge of class-specific adherence-resistance relationships may help clinicians and patients tailor therapy to match individual patterns of adherence in order to minimize the development of resistance and treatment failure. In addition, in low-income settings, this information may guide the selection of optimal drug combinations and regimen sequences to improve the durability of ART. Moreover, alcohol use and alcohol dependence are widespread in the general population. Many people suffering from alcohol use disorders also suffer from other psychiatric disorders including drug abuse disorders. Importantly, persons living with HIV should be assessed not only for their immunologic and virologic statuses, but also for comorbidities.

This is particularly important but rarely assessed or/and reported in the literature. Modeling or condition simulation may introduce these interactions in the context of the corresponding topic leading to possible interventions.

An important objective of our study is to bring awareness of these complex interactions in the medical and education fields. Awareness should lead to cooperation between patients living with HIV, their caregivers, and researchers looking into the mechanism of relationship between the virus, disease progression, alcohol, and its comorbidities. Multiple substances of misuse, such as combined alcohol and cocaine, might be associated with behaviour and metabolic consequences not measured or not considered in these analyses. These drug-induced biological phenomena may promote disease progression and CD4^+^ cell loss, as well as poor adherence with prescribed medication and/or inadequate micronutrient and macronutrient intake. Because the patterns of substance abuse observed in these HIV positive cohorts might not be common or typical of other HIV populations, these findings can be generalized only to other infected populations with similar patterns of substance abuse. Further studies targeting HIV heavy alcohol users, that control for other confounding behavioural and metabolic variables, need to be conducted to confirm and extend the knowledge in this area. Moreover, a network of direct discussion is needed between people living with HIV/AIDS, medical personnel treating HIV and/or addictions, epidemiology researchers, as well as policy makers and treatment planners.

## Figures and Tables

**Table 1:** Interactions between Alcohol and HIV Disease Progression.

Ref.	Study settings	Population characteristics	Alcohol use patterns	Main findings
			*Found an Association*	

[11]	USA	696 HIV positive patients	10.4% reported hazardous drinking (>14 drinks/week or >4 drinks/occasion for men and >7 drinks/week or >3 drinks/occasion for women)	Hazardous drinking associated with liver disease, defined as aspartate aminotransferase to platelet ratio index >1.5 (RR 3.72, 95% CI 1.40–9.87)

[12]	Italy	190 patients (71 HIV monoinfected, 53 HCV monoinfected and 66 HIV/HCV coinfected)		The extent of advanced liver fibrosis, defined as liver stiffness ≥9.5 kPa, correlate with alcohol intake (nonsignificant in HIV monoinfected patients, *P* < 0.001 in HCV monoinfected patients and *P* < 0.04 in HIV/HCV coinfected patients), but not with HAART exposure or duration of HAART

[13]	France	20940 HIV positive patients	Alcohol consumption of any kind in 25 (92.6%) of 27 patients who died from end-stage liver disease	Proportion of excessive alcohol consumption higher in 2003 compared to 1995 (*P* < 0.05)

[14]	France	24000 HIV positive patients	Excessive alcohol consumption (>30 g/day) reported in 23 (47.9%) of 48 patients who died from end-stage lived disease	The combination of alcohol and HCV coinfection led 12 (25.0%) deaths Consuming alcohol in excess of 30 g/day associated with death due to end-stage liver disease (*P* = 0.005)

[15]	France	210 HIV positive patients with a history of injectable drug use or HCV (60 HIV positive and 150 HIV-negative). There were 41 (19.5%) cases of liver cirrhosis	76 patients suffered from excessive drinking, with similar rate between HIV positive individuals and HIV-negative individuals	HIV positivity (OR 2.2, CI 1.1–4.5) and excessive drinking (OR 1.9, CI 1.0–3.9) independently associated with cirrhosis

[16]	Spain	2168 HIV positive patients, including 181 (8.3%) cases of cirrhosis	95 (52.5%) cirrhotic patients admitted current or past alcohol abuse	Alcohol consumption associated with cirrhosis (OR 3.5, 95% CI 2.5–4.8, *P* < 0.01)

[17]	Spain	91 HIV positive patients 30 (33.0%) patients suffered from liver toxicity 10 (11.0%) patients suffered from severe liver toxicity 43 (47.2%) patients coinfected with HCV and/or HBV		High alcohol consumption risk factor for liver toxicity (OR 3.35, 95% CI 2.43–4.62, *P* = 0.01)

[20]	USA	164 HIV positive patients	Patients consumed alcohol 88 (53.6%) were hazardous drinkers	Hazardous drinking associated with worsening of dyslipidemia (OR 3.18, 95% CI 0.99–12.05, *P* = 0.04)

[21]	USA	300 HIV positive patients, 82 (27.3%) patients experienced pneumonia	60% of sample reported prior or current alcohol abuse	Alcohol use independent predictor for pneumonia in HIV positive smokers (*P* = 0.004)

[22]	Spain	122 HIV-infected adults		Alcohol abuse independent predictor for bacteremic pneumococcal disease (OR 5.28)

[23]	Spain	25 HIV-1-postive patients with cerebrovascular ischemia		Cerebrovascular ischemia associated with history of high alcohol intake (OR 7.13, 95% CI 1.69–30.11, *P* = 0.007)

[25]	USA	72 HIV-negative light/nondrinkers, 70 HIV positive light/nondrinkers, 70 HIV positive heavy drinkers and 56 HIV-negative heavy drinkers	142 (53.0%) light/nondrinkers 126 (47.0%) heavy drinkers	Synergistic interaction between alcohol abuse and HIV infection with respect to motor and visuomotor speed

[26]	USA	31 male HIV positive patients, 27 patients with alcoholism, 43 patients with HIV infection and alcoholism comorbidity, and 22 normal healthy controls	70 (56.9%) patients with alcoholism	HIV and alcoholism comorbidity impair upper motor limb to a greater degree that HIV alone (*P* = 0.068) or alcoholism alone (*P* = 0.062)

[27]	USA	40 HIV positive patients, 38 alcoholic patients, 47 alcoholic HIV positive patients, and 39 controls	85 (51.8%) patients with alcoholism	Immediate episodic memory impaired in HIV positive patients with alcoholism comorbidity

			*Did Not Find an Association *	

[24]	USA	1539 HIV positive patients 881 (57.2%) reported HIV-associated sensory neuropathy, of which 335 (38.0%) reported neuropathic pain	845 (54.9%) had a history of alcohol abuse or dependence	History of alcohol abuse or dependence not associated with neuropathic pain caused by HIV-associated sensory neuropathy

[28]	Italy	76 HIV positive patients with bacterial community-acquired pneumonia 32 (42.1%) were receiving ART	25 (32.9%) alcohol abusers	Alcohol abuse not associated with a longer time before clinical stability was achieved

[29]	USA	299 HIV positive patients. Abnormal liver test results observed in 80 (26.8%) patients		Amount of alcohol consumed per week or alcohol overuse not predictors of liver test abnormalities

[30]	France	1175 HIV-infected patients 1048 (89.2%) were HCV coinfected		Alcohol consumption not associated with HCV-related serious adverse reactions

**Table 2:** The Role of Alcohol on the Immune System and the HIV Viral Load.

Ref.	Study settings	Population characteristics	Alcohol use patterns	Main findings
[33]	USA	220 HIV-1-infected IDUs receiving HAART	Heavy alcohol consumption (daily or 3-4 times per/week) reported in 139 (63.2%) patients. Men (OR 2.6, 95% CI 1.13–5.99, *P* = 0.013) and participants between 35 and 45 years of age more likely to be heavy alcohol users (*P* = 0.006)	Heavy alcohol consumption associated with 4 times lower chance of achieving undetectable viral load and 2 times higher chance of having a CD4^+^ cell count of <500 cells/*μ*L, compared to moderate alcohol consumption or abstinence

[34]	USA	595 HIV positive patients	245 (41.2%) subjects consumed alcohol	Heavy alcohol consumption associated with lower CD4^+^ cell counts only among subjects not on ART (*P* = 0.03)

[35]	USA	231 HIV positive drug users	126 (54.5%) participants consumed alcohol There were 53 (22.9%) frequent alcohol users (≥2 alcoholic drinks daily). No differences in alcohol consumption between patients on ART and patients not on ART	Frequent alcohol use (≥2 drinks/day) associated with CD4^+^ cell counts ≤200 cells/*μ*L (OR 2.907, 95% CI 1.233–6.855, *P* = 0.015). Frequent alcohol intake associated with higher viral load over time (*P* = 0.038)

[36]	USA	391 HIV positive patients	154 (39.4%) report past week alcohol consumption with mean number of 4 drinks 62 (15.8%) consumed >5 drinks/week	Consuming >5 drinks/week predictor for unsuppressed viral load (≥400 copies/mL) (OR 4.2, 95% CI 1.1–18.5, *P* = 0.046)

[38]	USA	2056 HIV-infected women and 569 HIV-uninfected women	33.6% of HIV positive women consumed ≥8 drinks/week 51.8% of HIV positive women consumed 1–7 drinks/week	Consuming ≥8 drinks/week related to higher risk of death (OR 3.39, 95% CI 1.54–7.44, *P* < 0.002)

[39]	USA	2702 HIV positive patients	Individuals were categorized as nondrinkers (no alcohol consumption), hazardous drinkers (consume ≥5 standard drinks on drinking days), and nonhazardous drinkers (consume <5 standard drinks on drinking days)	Nonhazardous alcohol consumption decreased survival by >1 year if frequency of consumption was ≥1/week, and by 3.3 years with daily consumption (from 21.7 years to 18.4 years). Hazardous alcohol consumption decreased overall survival by >3 years if frequency of consumption was ≥1/week, and by 6.4 years with daily consumption (from 16.1 years to 9.7 years)

**Table 3:** Alcohol Consumption and Nonadherence to ART.

Ref.	Study settings	Population characteristics	Alcohol use patterns	Main findings
			*Found Nonadherence *	

[36]	USA	391 HIV positive patients	154 (39.4%) report past week alcohol consumption, for a mean number of 4 drinks	At-risk drinkers (4 drinks/week for women and 5 drinks/week for men) are less likely to have current HAART prescription (*P* < 0.05). At-risk drinking a predictor for not being on HAART (*P* = 0.025)

[40]	USA	1074 HIV positive patients	315 (29.4%) patients presented with current or past history of drugs and/or alcohol abuse	Current or past history of drugs and/or alcohol abuse (OR 2.10, 95% CI 1.32–3.35, *P* = 0.002) and suboptimal adherence (OR 2.84, 95% CI 1.77–4.55, *P* < 0.001) predictors for virological failure

[42]	USA	43 HIV positive children	Alcohol abused by caregiver	Substance use by the caregiver associated with having higher viral loads in children patients (*P* = 0.007)

[43]	USA	197 HIV-infected individuals with history of alcohol problems who were receiving HAART	79 (40.1%) use alcohol	HIV positive drinkers less adherent to HAART than HIV positive alcohol abstainers (*P* < 0.05)

[44]	USA	1944 HIV positive patients	55% of 640 men and 28% of 1304 women consumed low levels of alcohol 15% of men and 8% of women consumed high levels of alcohol 7% of men and 4% of women engaged in binge drinking	Binge drinking (OR 1.75, 95% CI 1.17–2.64, *P* ≤ 0.05), moderate-to-heavy alcohol consumption (OR 1.47, 95% CI 1.08–1.99, *P* ≤ 0.05) and low alcohol consumption (OR 1.28, 95% CI 1.05–1.54, *P* ≤ 0.05) associated with nonadherence for women only

[45]	USA	82 HIV positive African-American patients		Alcohol can affect ART adherence through conscious decisions to skip medication while drinking and not through drunken forgetfulness

[46]	USA	5887 HIV positive patients	3573 (60.7%) respondents report alcohol use in past 12 months 630 (17.6%) alcohol users were nonadherent	Alcohol use in past 12 months associated with nonadherence (OR 1.3, 95% CI 1.1–1.5, *P* < 0.05)

[47]	USA	105 HIV positive patients without alcohol dependence	Mean monthly alcohol consumption was 4.64 ± 8.00 drinks/person	Monthly alcohol consumption associated with missed medication in the past 2 weeks (OR 1.08, CI 1.02–1.15, *P* < 0.01) and over the past weekend (OR 1.09, CI 1.03–1.15, *P* < 0.01) 47 (44.8%) patients missed a medication dose in the past 2 weeks, and 23 (21.9%) missed medication during the previous weekend

[48]	USA	275 HIV positive patients with alcohol use disorders 154 (56.0%) patients were nonadherent	An average of 84.9 standard drinks over the thirty days prior to the baseline interview	Alcohol consumption (*P* = 0.001) and number of drinks (*P* = 0.002) related to nonadherence

[49]	USA	1671 HIV positive women	60% of sample were abstainers and 26% were light drinkers (<3 drinks/week)	Light drinking (<3 drinks/week) (OR 1.51, CI 1.30–1.76, *P* < 0.01), moderate drinking (3–13 drinks/week) (OR 2.46, CI 1.96–3.09, *P* < 0.01), and heavy drinking (OR 4.37, CI 2.99–6.40, *P* < 0.01) associated with self-reported ART nonadherence

[50]	USA	67 HIV positive patients		Alcohol dependence is a specific and significant predictor of ART nonadherence in women only (*P* < 0.05)

[51]	USA	643 HIV positive IDUs		Fewer at-risk drinkers that nondrinkers reported receiving ART (OR 1.19, 95% CI 0.59–2.42)

[52]	USA	145 HIV positive patients	60 (41.4%) participants were current drinkers 11 participants (18% of drinkers) were problem drinkers (AUDIT score ≥8)	1 in 4 drinkers report stopping medication while consuming alcohol Alcohol use predicted treatment nonadherence (*P* < 0.05)

[53]	USA	335 HIV positive IDUs		Heavy alcohol use associated with first nonstructured treatment interruption (OR 1.58, 95% CI 0.92–2.70), early (within the first year) versus late treatment interruption (OR 1.55, 95% CI 0.51–4.73), and interruption of longer duration (≥6 months) (OR 3.21, 95% CI 0.83–12.5)

[54]	USA	1354 HIV positive women for whom HAART was indicated		Light drinking (OR 1.39, 95% CI 1.03–1.89, *P* ≤ 0.05), moderate drinking (OR 1.72, 95% CI 1.10–2.70, *P* ≤ 0.05) and heavy drinking (OR 2.29, 95% CI 0.96–5.47) associated with nonadherence, compared to nondrinking

[55]	USA	224 HIV positive patients	Baseline prevalence of past year hazardous drinking was 27% (AUDIT score ≥8)	Hazardous drinking associated with nonadherence

[56]	France	445 HIV positive patients	329 (73.9%) patients consumed ≤1 unit of alcohol/day at baseline 116 (26.1%) patients consumed >1 unit of alcohol/day at baseline	Baseline alcohol consumption associated with nonsignificant nonadherence after 4 months (*P* = 0.09)

[57]	France	276 HIV positive IDUs receiving HAART	Approximately 84% of patients report alcohol consumption during the past 6 months	Monthly alcohol consumption during past 6 months associated with ART nonadherence (OR 1.15, CI 1.08–1.23, *P* < 0.001)

[58]	France	1010 HIV positive patients	59 (5.8%) patients report daily alcohol consumption	Nonadherence more common among subjects who consume alcohol daily (OR 0.39, CI 0.20–0.58, *P* < 0.001)

[59]	France	2340 HIV positive patients receiving HAART. Harmful alcohol consumption was frequent	12% of patients had symptoms of potential alcohol abuse/dependence during the previous 12 months (CAGE questionnaire score of ≥2) 27% of patients suffered from hazardous drinking or alcohol use disorders (AUDIT-C questionnaire score of >4 for women and >5 for men) 9% of patients reported regular binge drinking (≥6 alcohol units drunk consecutively at least twice a month)	Harmful alcohol consumption associated with nonadherence to HAART (*P* < 0.001) for regular binge drinking and symptoms of alcohol abuse or dependence

[60]	Switzerland	6709 HIV positive patients		Increasing alcohol intake associated with deteriorating adherence to ART (OR 1.25, 95% CI 1.10–1.43)

[61]	Sweden	946 HIV positive patients	15.5% of patients report alcohol and drug problems	Adherent patients more likely not to have problems with alcohol (OR 1.8, 95% CI 9 1.18–3.01, *P* = 0.008)

[62]	Australia	1106 HIV-infected patients 867 (78.4%) report taking cART, 339 (39.1%) of which report difficulty adhering to medication		Alcohol use associated with self-reported nonadherence (OR 1.47, 95% CI 1.03–2.09, *P* < 0.05)

[63]	South Africa	12 HIV positive patients receiving HAART		Alcohol abuse identified as barrier to adherence

[64]	South Africa	8 male HIV positive patients		Patients delay HIV treatment while coping with alcohol dependence

[65]	South Africa	56 HIV positive children		Alcohol use by caregiver associated with poorer ART adherence in children patients (*P* < 0.01)

[66]	Cameroon	533 HIV positive patients	60 (11.3%) patients reported binge drinking	Binge drinking associated with interruption of ART

[67]	Ethiopia	422 HIV positive patients	31 (7.3%) subjects report alcohol consumption, 6 of which did so on a regular basis	Alcohol drinking associated with nonadherence (OR 0.210, CI 0.071–0.617, *P* = 0.003)

[68]	Botswana	300 adult HIV positive patients		Alcohol use predicted poor ART adherence (*P* < 0.02)

[69]	Benin, Côte d'Ivoire, and Mali	2920 HIV positive patients		Current drinking (OR 1.4, 95% CI 1.1–2.0), especially hazardous drinking (OR 4.7, 95% CI 2.6–8.6), associated with nonadherence

[70]	Brazil	306 HIV positive patients	37.6% of sample consumed alcohol in month prior to baseline interview	ART nonadherence associated with alcohol use in month before baseline interview (OR 1.61, 95% CI 1.08–2.39, *P* = 0.018)

[71]	Brazil	295 HIV positive patients	109 (37.3%) subjects consumed alcohol in month prior to baseline interview	Nonadherence to ART associated with alcohol use (*P* < 0.001)

[72]	Thailand	205 HIV positive patients	13 (6.3%) subjects report current alcohol use	Current alcohol use sole predictor of nonadherence to HAART (OR 1.67, CI 1.05–2.48, *P* < 0.001)

[73]	India	198 HIV-infected patients receiving HAART		Alcohol use associated with nonadherence (OR 5.68, 95% CI 2.10–15.32, *P* = 0.001)

*Did Not Find Nonadherence *				

[74]	USA	1030 HIV-infected women		No delay in ART initiation between heavy drinkers and nondrinkers

[75]	USA	300 HIV positive men who have sex with men	43% of sample report alcohol consumption in first 2 weeks post-baseline	No association found between alcohol use and nonadherence

[76]	UK	394 HIV positive patients		Excessive alcohol consumption borderline significantly lower in patients receiving HAART (*P* < 0.08)

[77]	Uganda	2311 HIV positive patients 928 (40.2%) presented late for treatment	123 (5.3%) used moderate levels of alcohol and 360 (15.5%) used high levels of alcohol	Alcohol consumption in past year (assessed using AUDIT-C) negatively associated with late presentation for treatment (OR 0.65, 95% CI 0.44–0.96, *P* = 0.03 for moderate use and OR 0.79, 95% CI 0.61–1.00, *P* = 0.05 for heavy use)
